# ‘My dog is my life’: One Health approaches to healthcare for people and their companion animals experiencing homelessness in the United Kingdom

**DOI:** 10.1371/journal.pone.0354517

**Published:** 2026-09-03

**Authors:** Rebekah Sullivan, Andrew Gardiner, Glen Cousquer

**Affiliations:** 1 Royal (Dick) School of Veterinary Studies, University of Edinburgh, Easter Bush Campus, Roslin, Midlothian, Scotland; 2 Working Animals International, 55 Ludgate Hill, 2nd Floor, London, United Kingdom; University of Minnesota, UNITED STATES OF AMERICA

## Abstract

Around one in ten people seeking shelter from homelessness in the United Kingdom have a companion animal, most commonly a dog. Dog ownership when homeless can pose a barrier to accessing healthcare and accommodation, and may force individuals to sleep on the streets. People experiencing homelessness with a dog are therefore a stigmatised minority within a minority, often invisible to support services and hard to reach. The bond between human and canine in these situations is usually strong; the dog is often the most significant ‘other’ in that person’s life. Failure to accommodate this relationship as part of a holistic approach has implications for both human and animal healthcare in the homeless community. Bringing these two healthcare disciplines together represents a specific application of One Health that can help us better realise their collaborative transdisciplinary potential. Interpretative phenomenological analysis was used to explore health needs and challenges for people experiencing homelessness with companion dogs in the UK and to investigate the feasibility of joint healthcare provision. Study participants included people experiencing homelessness and service providers working in veterinary or human healthcare and housing services. Human and animal health were considered entangled by both service users and service providers, requiring an approach which accounts for the needs of human and animal together. The idea of joint delivery of human and animal healthcare at the same venue was of interest to both service users and providers but also raised concerns. While sparse examples are available in the literature, a more workable concept may be to aim for a ‘coordinated multi-species healthcare’ which is responsive and empathic to the needs of both people and dogs.

## Introduction

Following its first meeting in 2021, the newly formed One Health High Level Expert Panel (OHHLEP) set out a definition of One Health, underpinned by five foundational principles [1,2]. There is now substantial familiarity with the basic tenets of One Health: that the health and wellbeing of humans, animals and environment are interdependent and form an ecosystem. The OHHLEP foundational principles (Box 1) provide a call to action to promote wider and deeper One Health thinking. This paper responds to that challenge by examining the One Health concept at an intensely local level, recognising that this potential for One Health is all-too-easily lost among global needs for effective international disease surveillance, food security and responsible use of antimicrobials [3]. Localised studies can hold important lessons for how to make the One Health concept applicable and relevant to everyday interactions between humans, animals and the ecological processes that sustain them. The site of interest in this paper is the urban ecosystem, with its stark geographies of inequality; we are interested in exploring One Health at ’street level’.

**Box 1: Foundational principles (FPs) of the OHHLEP.**

- FP1: Equity between sectors and disciplines.
- FP2: Socio-political and multicultural parity (the doctrine that all people are equal and deserve equal rights and opportunities) and inclusion and engagement of communities and marginalised voices.
- FP3: Socio-ecological equilibrium that seeks a harmonious balance between human-animal-environment interaction and acknowledging the importance of biodiversity, access to sufficient natural space and resources, and the intrinsic value of all living things within the ecosystem.
- FP4: Stewardship and the responsibility of humans to change behaviour and adopt sustainable solutions that recognise the importance of animal welfare and the integrity of the whole ecosystem, thus securing the well-being of current and future generations.
- FP5: Transdisciplinarity and multisectoral collaboration which includes all relevant disciplines, both modern and traditional forms of knowledge, and a broad representative array of perspectives.

This study concerns the application of One Health in marginalised groups of humans and their companion animals. Cheraghi-Sohi et al [4] describe marginalised groups as ‘populations outside of mainstream society’. Marginalised may also be referred to as ‘under-served,’ ‘hard-to-reach’ or ‘vulnerable’. Our marginalised humans are people experiencing homelessness (PEH) in United Kingdom (UK) locations. PEH retain essential human needs of belonging, connection and companionship, needs which can be met through relationships with companion animals (we avoid the term ‘pets’ because this word does not capture the depth and significance of the human-animal bond in this and other contexts). In marginalised communities, humans and companion animals have health and well-being needs that are deeply entangled and in danger of going unacknowledged and therefore unanswered. We set out to explore these joint healthcare needs, which represent an opportunity to practise One Health and to address health inequities [5] and inequalities [6,7].

In North America, the concept of providing joint healthcare to marginalised communities has been realised through student-led clinics in single-location buildings delivering veterinary and medical services to companion animal and owner [8,9], or through provision of veterinary services at established human healthcare practices [10–12]. In this study, we sought to investigate whether there was scope for similar One Health models in the United Kingdom.

The study described here is not focused on the general health and psychological benefits of the human-animal bond, which have been well described (e.g., [13,14]). Instead, we explore the challenge and feasibility of joint healthcare provision to meet the needs of both human and animal in PEH. The overall aims of the study were two-fold. First, the linked healthcare needs and challenges of people and animals in homeless communities in six UK locations were identified. Secondly, as no formalised One Health clinics or centres similar to those models in published examples from North America currently exist in the UK, the concept of joint healthcare was discussed with participants. This allowed us to explore with participants the possibility and relative merits of joint healthcare provision, identifying what is, or could be, successful and what barriers exist. The concept of joint healthcare thus acted as a useful lens through which to approach One Health dimensions of care as currently delivered to PEH. This study acknowledges One Health FP2, recognising that those experiencing homelessness in the UK are at great risk of marginalisation and social exclusion. FP1 and FP5 call for equity between sectors and disciplines, striving for a transdisciplinarity that breaks traditional disciplinary boundaries and allows emergent synergies to be realised through collaboration. In addition to exploring existing collaboration between veterinary and human healthcare professionals, we sought to engage service users and service providers so as to better integrate insights and align these with a common aim: to deliver improvements in health and well-being for human and animal together.

## Research approach

### Participant recruitment

Participants were recruited purposefully [15] from different geographical locations and backgrounds. Purposeful/purposive sampling ensured that:

1. Willing participants, able to provide insight and share experiences, were recruited.
2. Risks posed by the sensitive nature of the topic were minimised by identifying service users who would not have their mental health compromised through participation. This identification was facilitated on the day of interview, by the hostel manager and staff who conferred to decide which residents were currently well enough to participate.

Service provider participants were identified using ‘snowball sampling’ [16] from initial research team contacts. Tables 1–3 describe participants and inclusion criteria.

**Table 1:** Study groups and rationale for inclusion.

Group	Stakeholder status	Rationale for inclusion
Service providers	Veterinarians	• Provide preventative healthcare and treatment of companion animals • Understand the common clinical conditions of companion animals belonging to PEH and logistical constraints in providing care
	Veterinary nurses (‘vet techs’ in the US)	• Provide veterinary support with a focus on nursing care
	Medical doctors	• Provide preventative healthcare and treatment of human patients • Understand the common clinical conditions of PEH and logistical constraints in providing care
	Medical nurses	• Provide nursing perspective on needs of PEH • Identify healthcare needs requiring doctor input
	Housing shelter/hostel staff	• Have a non-medical relationship with PEH and their animals and insights into social care and wellbeing • Provide perspectives on how to accommodate animals in support services
Service users	People experiencing homelessness (PEH) and their companion animals	• Account for their own health and social care needs and their experiences of services • Discuss the relationship with their companion animal and need for veterinary support • Offer insight into future provision of health and social care services for themselves and/or their animal

**Table 2:** Criteria for inclusion in the study. Service users were excluded if there was any concern about their mental or physical health, as raised by the hostel staff. Service providers were excluded if they were not proactively involved with provision of animal or human healthcare and social services to people experiencing homelessness.

Stakeholder group	Criteria for inclusion
Service users	• Currently or previously homeless or vulnerably-housed • Currently own a companion animal or have done so at the same time as being homeless or vulnerably-housed **The three service users recruited came from a single homeless hostel in Edinburgh**
Service providers	• Currently working with or having recently (no more than 18 months elapsed) worked in health or social care for PEH • Participants did not have to have had experience of working with PEH specifically with companion animals: the aim was to gain a broad understanding of health and social care provision and needs of this human population experiencing homelessness. **The service providers recruited came from different charitable organisations, NHS Trusts and geographical locations**

**Table 3:** Summary of participant data. All participants were given a pseudonym. Time in sector is included to indicate level of experience. The three service users had a companion dog who accompanied them during the interviews.

Participant pseudonym	Location	Role of participant (abbreviation in text)	Age	Gender	Time in sector
Andrea	Glasgow	Veterinarian (Vet)	30-39	Female	3 years
Bridget	Suffolk	Veterinarian (Vet)	50-59	Female	10 years
Jack	Edinburgh	General practitioner (GP)	50-59	Male	15 years
Sally	Exeter	General practitioner (GP)	40-49	Female	10 years
Carla	Edinburgh	Housing professional (HP)	30-39	Female	1 year
Beatrice	Edinburgh	Service user (SU)	20-29	Trans	N/A
Chris	Edinburgh	Service user (SU)	20-29	Male	N/A
Steve	Edinburgh	Service user (SU)	30-39	Male	N/A
Karl	Edinburgh	Housing professional (HP)	40-49	Male	21 years
Lucy	Edinburgh	Community psychiatric nurse (CPN)	50-59	Female	7 years
Heidi	Midlands	Animal welfare professional (AWP)	20-29	Female	5 years
Jane	Plymouth	Veterinary nurse (VN)	40-49	Female	4 years
Tammy	Edinburgh	Veterinary student (VS)	20-29	Female	< 1 year

### Ethics and consent

The study was approved by the Human Ethical Review Committee of the Royal (Dick) School of Veterinary Studies, University of Edinburgh (HERC_795_21), thereby ensuring that the research conducted aligns with relevant guidelines and legislation. Participants were provided with an Information Sheet and Consent Form, allowing written consent to be obtained. No incentives or benefits were offered to participants.

### Participant interviewing

Single interviews lasting 1–2 hours took place during February to April 2022 and were conducted either online or at a location of the participant’s choosing. Initial questions determined sample demographics. Thereafter, a semi-structured interview was used to explore the central research questions:

- What healthcare needs and challenges do people experiencing homelessness (PEH) and their companion animals encounter in the UK?
- Is joint healthcare provision (for human and animal) desirable and feasible?

### Data processing and analysis

Interviews were recorded and auto-caption transcripts generated. Transcripts were reviewed against the recordings and edited in Microsoft Word to correct transcription errors.

Interpretative Phenomenological Analysis (IPA) was selected for data analysis as best suited for exploring peoples’ lived experiences of a specific phenomenon [17]. IPA’s double hermeneutic approach, in which participants provide their own interpretation of their experience which the researcher in turn interprets, is considered appropriate for ‘topics which are complex, ambiguous and emotionally laden’ [18]. The number of participants in this study (*n* = 13) exceeded that typically used for an IPA project, with Smith el al 2022 [17] recommending *n* = 3–10. Small sample sizes are desirable to enable the detailed idiographic analysis of lived experience using IPA. Participants are selected to be representative of a perspective, rather than a population. The larger number of participants here is not, however, a drawback as they are split into the two sub groups of service user and service provider. This further recognised use of IPA [17] delivers multiple perspectives through tightly focussed interviews around the central research questions, including when individuals from a larger sample size and seemingly disparate groups are connected by sharing important characteristics, in this case the different relationships with homelessness, be this personal or vicarious. Therefore, this multi-perspective study compares and contrasts the IPA of service providers and service users in order to gain a broad and holistic understanding of the healthcare needs and challenges of the specific population studied.

Initial annotations were made on each transcript to identify recurring ideas related to the research questions. This annotation process was repeated several times to ensure data immersion and capture of all relevant ideas. Using the process outlined by Smith, Flowers and Larkin [17], these coding annotations were then used grouped into themes for each participant (personal experiential themes − PETs). Group experiential themes (GETs) were subsequently elicited from amalgamation of PETs from both service users and providers, the selected GETs having been referenced by over half of participants. JARS-Qual table guidelines [19] were followed to ensure that the process was systematic and reliable.

### Researcher positionalities

All three researchers are veterinarians who have extensive experience of working in charitable sectors and with marginalised communities ‘on the ground’. Whilst no researcher has lived experience of homelessness, both RS and AG provide veterinary support to animals and their humans experiencing homelessness in the UK. RS conducted all interviews and has only provided volunteer veterinary support in the south-west of England and not for the duration of this study, therefore mitigating against bias and any chance of knowing the service user population, who were recruited in Edinburgh, Scotland.

## Results

### Research question 1: Healthcare needs and challenges

Group experiential themes were identified for both healthcare needs and healthcare challenges. There is some inevitable repetition of themes between needs and challenges, as shown in the theme table (Table 4).

**Table 4:** Group experiential themes (GETs) for healthcare needs and challenges.

	GETs
**Healthcare needs**	Maintaining the human-animal bond
	Animal healthcare
	Acknowledging a background of trauma
	Need to build trusting relationships
**Healthcare challenges**	Stigmatisation
	Accessing healthcare
	Third sector involvement
	Animals as barriers
	Inconsistencies in care

### Healthcare needs

(i) Maintaining the human-animal bond

Maintaining the human-animal bond was an absolute necessity for service users, for whom dogs were seen as family, guardian angels, best friends, but never ‘pets.’ Most homeless hostels do not permit dogs, forcing service users to make a choice:

Steve (SU): ‘I would rather go back on the streets sleeping rough than lose her because then [*going into a hostel*] I will be by myself.’

For Chris (SU), being able to provide for his dog brought a sense of peace and stability:

‘I think they help people so much with mental health and loneliness and companionship. She’s pulled me through*.*’

Dogs provide motivation to get up each day, to take exercise and to care for another’s needs, which in turn raises self-esteem and boosts mood. Service providers, too, were cognisant of these positive influences:

Lucy (CPN): *‘*Dogs are with these people 24/7. That’s their life. That’s a protective factor, who they have relationships with, and you can’t split them.’

Allowing patients to bring their dogs to a medical check-up can revolutionise the experience for provider and user:

Sally (GP): ‘The whole appointment, he wasn’t intimidating, or aggressive. He didn’t accuse me of anything which is how the other appointments had been going. It was amazing how having the dog there diffused any tensions.’

This comment from Sally illustrates the relief that can arise, for both parties, when the service user is allowed to bring their choice of chaperone, in this case a canine rather than human companion. In all human medical care appointments, patients can exercise their right to bring a chaperone. For those humans who trust no-one other than their companion animal, facilitating an animal-inclusive appointment policy can improve the outcome of any interaction–for both doctor and patient.

(ii) Animal healthcare

All three companion dogs appeared in excellent physical health. However, PEH will often say that their dogs show signs of separation anxiety if left alone and all three service users in our study reported this. Separation anxiety is often constructed biomedically as a ‘behaviour problem’ in veterinary medicine. A less anthropocentric view is that separation anxiety is a natural response to an unnatural situation for social canids. The dogs of PEH are rarely left alone so it is not surprising that when this happens, it may cause distress. It should be noted that separation anxiety may manifest in the separated human too.

While studies show that dogs belonging to PEH generally have happy and healthy lives [14], service providers acknowledged that this is not always the case:

Sally (GP): *‘*It’s not universal that people who are homeless look after their pets. They often are the most important thing in that person’s life, and very treasured. But there are instances where there’s been real neglect going on.’

Service providers understood that those who struggle to take care of their own well-being may sometimes fail to provide adequate care for animals, however loved.

It was notable that PEH often purposefully seek out preventive healthcare for their companion animals:

Andrea (Vet): *‘*I was pleasantly surprised how proactive the population was in terms of coming regularly, just to make sure that their dogs were getting their wormers, flea treatments, vaccinations.’

Such engagement with preventative care provides opportunities to build connections and trust, which can help deliver longer term benefits for both animal and human health and well-being.

(iii) Acknowledging trauma

Service users hinted at traumatic experiences and ongoing mental ill-health challenges: Chris escaped domestic abuse; Beatrice fled trans-hate in her hometown; Steve acknowledged alcohol dependency and bore multiple self-harm scars.

Service providers were unanimous that trauma is significant in the past and present of all PEH they encountered and thus a major healthcare issue:

Karl (GP): ‘Homelessness − 99.99% is not just a bricks and mortar issue. It comes with a plethora of issues. Every single person who comes through the service has suffered some degree or another of trauma.’

According to the experiences of these service provider interviewees, every PEH they encountered also had substance abuse concerns related to previous trauma.

Lucy (CPN): ‘A hundred per cent of my caseload have trauma. I don’t know why they would want to come off drugs, to be honest.’

These service provider observations reflect a sense of frustration when faced with patients denied access to healthcare services due to concurrent substance abuse. The service providers interviewed were adamant that the substance abuse was intricately bound up with the history of trauma; both substance abuse and trauma were thus major barriers to accessing healthcare.

(iv) Building trusting relationships

Use of the word ‘trust’ by service providers paralleled that of trauma. Meaningful and ongoing engagement with health services presupposes trust. Trust is built through first identifying what is important to an individual and suspending judgement and cynicism [20]. Jack (GP) recognised that validating a PEH’s relationship with their dog can help forge a trusting connection:

‘You’ll see folks that take what we would see as terrible neglect for their own wellbeing but will totally look after their dogs. And if you meet them at that point, then maybe trust develops.’

Lucy (CPN), too, had grasped the opportunity for developing trust afforded through acknowledging someone’s animal:

‘And that’s where they trust that you were OK because you liked the dog. For me to be there to connect, get someone to the practice, get them registered, getting them all their healthcare − just from speaking to them about their dog.’

Good veterinary care can also be achieved through demonstrating commitment and building trust:

Jane (VN): *‘*When we go out, we wear hoodies, because it immediately makes us more approachable. It’s important to encourage them to come back for the flea and worm treatment and their cuddles and their food. Because when the dog gets sick, we don’t want to be the police or the social worker. We just want to be [*the person who*] is going to look after their dog or their cat because it’s important.’

Jane recognised that the simple action of wearing everyday clothes rather than a uniform can make a health worker more approachable and, through providing preventative care and supportive gestures, trust can be built.

### Healthcare challenges

(i) Stigmatisation

Inadequate healthcare provision frequently arises from conscious or unconscious stigmatisation by professionals and public. Sally (GP) experienced use of destructive, language towards patients:

‘My patients, they tell me, “The nurse called me a street rat and so I left [*hospital*].”

I'm like, Why did you self-discharge?

“Because they told me I was a waste of space or I’m using up a bed”.’

Sally contemplated the frustration of others at a perceived disparity/reverse bias in service provision, remembering an angry policeman asking why the medical team were talking to PEH on the street:

*‘*The policeman said, “Why should a GP be finding patients here when no one else in Exeter can get an appointment?” You can see the conflict that arises for people.’

Jack (GP) believed stigmatisation was portrayed by structural inequalities in healthcare funding:

*‘*It totally bears out the inverse care law. People that need it [*healthcare*] least get the most and people who need it most get the least. And you see that massively regarding people in homelessness. And people in homelessness are *the* *poorest*.’

The ‘inverse care law’ was first identified by Hart in 1971 [21] but is still, according to Jack, as relevant today. A marginalised sector of society has significant healthcare needs, but a minority of these needs are being fully met.

(ii) Accessing healthcare

In contrast to what was said by service providers, the three service users stated they had no issues accessing healthcare, although Chris refused medical care if he couldn’t take his dog along with him:

‘They always used to say, “Don't bring the dog with you.” So I used to miss my appointment.’

Chris feared medical professionals insisting his dog was removed or otherwise losing his dog while he was attending an appointment (e.g., if stolen while tied up outside a clinic):

‘People get quite scared of leaving the dog because it's the only thing they've got.’

Improving access to healthcare is commonly addressed through provision of outreach services in which service providers go to the patients. For Sally (GP), outreach is primarily about engaging, starting along the road to trust:

‘What we do in health outreach is not to treat [*or*] diagnose. It's to start a conversation and say, “Let's look at that hand, that looks like it needs dressings on. Why don't you come, and I will see you at the surgery in half an hour?”’

However, outreach provision is complex, posing practical challenges and necessitating a leap into unfamiliarity territory for some providers. Jack (GP) thought many medics were apprehensive about outreach, which may come from feeling physically and professionally insecure in non-clinical environments:

*‘*People are often very reluctant to do outreach as actually for healthcare stuff, it's a lot more challenging. You're out of your own comfort zone. It is resource intense.’

There are inevitable logistical challenges and constraints associated with outreach, whether medical or veterinary:

Andrea (Vet): ‘You're limited in your practical supplies that you can carry. There are some things that are just completely outside the remit of a street-based veterinary service.’

Sally (GP): *‘*There are limitations to how much you can examine someone because of [*the need to protect*] their privacy and [*what topics*] you can talk about, especially if they're in a crowd.’

These two comments highlight both the limited practical resource capacity available for ‘on the street’ medicine and the more off-putting challenge of lack of privacy – a factor that needs careful consideration for wary, distrusting individuals. Sally’s earlier comment regarding the role of outreach as a bridge builder and introduction to services demonstrates how such logistical challenges need not be a barrier to further support.

(iii) Third sector involvement

The third sector (non-governmental and non-profit making organisations) plays a key role in meeting gaps in healthcare provision to PEH. Service providers described the benefits of third sector involvement whilst also highlighting concerns. Reliance on charitable donations is precarious yet remains critical for sustaining services:

Sally (GP): ‘Without all the public support for these big charities, support available for people who are homeless would certainly be much less.’

Staff turnover in third sector organisations is high, negatively impacting the ability to build trusting relationships between provider and user:

Sally: ‘Charities are often small and have tight margins. They don’t pay their staff that well, so they find it hard to retain.’

#### This inherent unpredictability has knock-on effects on service users

Jane (VN): ‘St——[*local charity*] has been doing a feeding scheme for two years, which we've had one of our clients on. And then they ran out of money, so they stopped it. There's no consistency.'

The precarious nature and inconsistency of support risks further alienation of service users, who may baulk at the challenge of building trust with providers that may not be able to go the distance if their funding fails.

(iv) Animals as barriers

Animal ownership may create difficulties with employment, healthcare and housing access for PEH. Major concerns arise when animal owners require hospital treatment:

Sally (GP): ‘She [*service user*] is in this vulnerable position, needing residential rehab but refusing to go in and leave the dog. But equally, there's times when she's been suicidal, and she wouldn't kill herself because of the dog.’

This situation, echoed by other service providers, illustrates the conflicts that can arise between human and animal health needs. Some PEH will not attend in-patient care because there is no one they trust to look after their animal.

Service users also alluded to animal ownership preventing work opportunities. However, Jane (VN) considered how this could be reconciled through improved mental health:

*‘*I just think, if you help them be more mentally stable and raise their self-esteem, then they can find ways around finding somebody to look after the dog like the same as any of us. I don't think the dogs are barriers to getting work. I think mental health is the barrier to getting work*.*’

This comment questions dog ownership invariably perceived as a barrier: if acknowledging the dog acts as a conduit to building trust with health workers, then the dog can actually be a route to improved mental health. With improved menthal health, other possibilities are opened up, including independent living and employment.

(v) Inconsistencies in care

For many different reasons, service users are inconsistent in attending for healthcare:

Sally (GP): ‘They come in once and then we don't see them for months, or they move then they'll come back to a place. It is very difficult to have continuity.’

Inconsistencies in accommodation and social care provision were evident when comparing Edinburgh, where there are hostels which allow dogs, with East Anglia, where there were none at the time of the study:

Bridget (Vet): ‘One of the major issues that we've got here is that temporary emergency housing does not allow animals, even though the accommodation could cope.’

These inconsistencies signify the difficulty that would arise in trying to find one solution that fits across the UK and suggests national advocacy is needed to address disparities in care provision.

### Research question 2: Exploring joint healthcare

Transcript analysis allowed construction of four themes relating to joint healthcare:

- Demand for joint healthcare
- Examples of joint healthcare
- What could joint healthcare include?
- Perceived barriers to joint healthcare

(i) Demand for joint healthcare

Veterinary and animal welfare workers often commented on the need to provide health support for animal owners. Heidi, whose role was to help identify suitable accommodation for PEH with dogs, recognised the implicit health connection:

‘Because we are supporting their dog, we often end up supporting the person as well. I think that they kind of have a different level of trust with us. We are there for their dog and that's so important to them.’

Bridget (Vet) believed animal health workers who had established trust and rapport with PEH could help organise temporary animal care, which would then allow the owner to accept in-patient treatment, knowing their animal was safe:

‘My vision is to have areas in community centres where people can go, somewhere someone can come and say, “I've got to go into hospital in three months’ time. Could you help me with finding a solution to look after my pet?”’

When service providers form trusting relationships with service users, joint healthcare can become a reality:

Jane (VN): ‘Once you care for some of these animals, and you show that you can care for something without judgement and without expectation, then you start breaking down these barriers that they've put up, and then through their animals, you can care for them a little bit like just making sure they're OK, or give them a phone number of someone to look after them.’

Veterinarians and veterinary nurses were held in high regard by service users, which puts them in a unique position to help facilitate joint healthcare:

Chris (SU): ‘I can’t praise them [*vets and vet nurses*] enough.’

Vets can draw on the trust and respect gained through animal treatment to tactfully point service users towards appropriate human healthcare.

(ii) Examples of joint healthcare

The possibility of joint healthcare emerges naturally in settings when animals and humans are in proximity with pressing needs to be addressed. Inter-professional cooperation takes place in the context of addressing practical issues and problems. Prior networking can potentiate this useful exchange and increase the benefits to both animals and humans. Heidi, an animal welfare professional, maintained good communication channels with local GP practices:

‘We may need to speak to them [*GP practice*] about a specific case if we're not able to get hold of the dog owner. We do have a couple of GP practices registered with us. They'll refer clients on to us too.’

Likewise, help for an animal may come about from appropriately informed human healthcare providers:

Sally (GP): ‘We've had people who've got mental health problems, neglecting their pets. Health workers have gone in to see them because we're worried about their health and picked up that their dog is not being treated well. Normally someone in our outreach team will know who to contact if there's a dog that's in distress. They will have the links, know how to get that dog the treatment it needs.’

As Sally highlights, transdisciplinary awareness and collaboration will lead to improved health outcomes for human and animal.

(iii) What could joint healthcare include?

Bridget (Vet) was proud of a network of human and animal health professionals she had built which allowed a coordinated approach to joint healthcare and which led to training opportunities and the sharing of knowledge and experience:

‘I prescribe my knowledge and my network to help advocate and find solutions and break down barriers. […]

We do always look to see what new training is available that will help someone when they are supporting people.’

Bridget appreciated that service providers themselves need support, particularly when outside their professional comfort zone. Bridget’s charity initiated a unique approach to joint healthcare, securing funding for an ‘animal companionship practitioner’ post in the local area. The aim of the post is to liaise between human and animal healthcare professionals, whether in statutory, private or third sector services:

‘We want to use our knowledge to create animal-centric people in human health and social care and human-centric people in the veterinary world. We’ve established this role of an animal companionship practitioner, providing animal companionship support services and utilising our links with other community, statutory or voluntary sector services.’

Karl (HP) had valuable experience of the multifaceted needs of service users as a hostel manager and was positive about the potential of joint healthcare. Through understanding where service users are starting from, Karl could envisage how, at one level, joint care may simply consist of professionals co-ordinating how they provide an approachable service for both human and animal*:*

‘I think it [*having medical outreach teams alongside vets*] is very workable. Even if it is just that contact, that initial, “We're here - do you know about us?” I mean these conversations help people to engage with the services, you know?’

Lucy (CPN) wished to promote the placing of human healthcare professionals within veterinary services and vice versa and cited examples locally:

‘I came up with this idea. If we could get this veterinary clinic into the practice [*local homelessness health servic*e] once a month or something and I had a presence at their [*veterinary outreach*] clinic and not make it about anything clinical. Could it be a joint venture that would encourage people?’

Lucy had recognised that, outside of an emergency, care does not need to start with addressing clinical needs but with opening the door to service providers, through building awareness and trust from the service users.

(iv) Perceived barriers to joint healthcare

Interview participants cited multiple examples of service users avoiding static venues. Karl (HP) advised that some PEH fear meeting acquaintances in established healthcare settings:

‘The homeless community is quite small, and they know each other, and they’ve got gripes with each other. They don't want to see these people so it’s a barrier to accessing healthcare.’

Showing patience with service users, and appreciating that official venues may feel threatening, was seen as essential:

Sally (GP): You've got to really persist to get people to come to you. That's why it's so important, to go to people, not expecting them to come to a static place that's quite intimidating for them.’

There were conflicting opinions about combining human health and social care services under one roof. Lucy (CPN) was impacted by the strength of feeling expressed by one service user who had been to a multi-agency hub:

‘They felt ghettoised by having all these services in one place, really.’

This made Lucy question her own enthusiasm for combined approaches.

Covid vaccination clinics in Devon presented a small-scale example of joint healthcare in action. A national veterinary service supporting PEH worked alongside the vaccination clinic, providing veterinary support whilst service users received their covid vaccinations:

Jane (VN): ‘It worked quite well last year because our clients came to get vaccinated. They liked the fact that they could leave their dogs with us and go in and get seen.’

But Jane, like Lucy, believed that combining too many services can have negative consequences for some service users:

‘Some of them didn't come because they also had housing there. And they also had the dentist. And they didn't want to see them and deal with that. They just wanted to get their jab.’

Jane’s interpretation reveals that the inclusion of multiple services under one roof can be overwhelming, to the extent of being off-putting, but some service users liked combined services. Chris (SU) was especially keen about the prospect of a ‘one stop shop’ for animal and human care:

‘Fantastic, kill two birds with one stone. I think that’s brilliant because you could meet other new people that's in the same situation as yourself.’

Sally (GP) also relished the efficiency and resource-saving nature of single access points even as she also (as quoted above) recognised the advantages of taking services out to the users:

‘My building is a hub of all charities that are involved with the population. It's an amazing place to be because if I need housing advice for one of my patients I can just walk through a door.’

The issue of combined service provision is therefore interesting, with practical advantages and synergies, as well as hidden social and interpersonal aspects which may be off-putting to some service users who, perhaps, prefer to deal with only one issue at a time.

## Discussion

This study set out to identify the healthcare needs and challenges of PEH and their companion animals and to explore the desire for and feasibility of novel, joint human/veterinary healthcare approaches in the UK context. The multi-perspective participant narratives in this study identify some key healthcare needs and challenges for PEH who wish to sustain their crucially important and stabilising relationship with a companion animal. This relationship is all too easily erased by health and social providers who set out to help PEH but fail to see the animal as a ‘most significant other’. It is the existence and quality of this relationship that underpins meaningful One Health in these under-served communities.

The complex circumstances revealed in the rich narratives of homelessness in this study are challenging for all but provide an excellent opportunity to reflect upon what is needed to make ‘one healthcare’ more accessible. The ubiquitous background of trauma for PEH requires professionals to focus on building rapport such that service users can begin to trust and engage with services. PEH with animals are at great risk of stigmatisation from the public and even from the professionals whose job it should be to help them. Service providers who recognise the significance of the human-animal bond see the companion animal as a way to ‘reach in’ empathically to a homeless person’s life. The companion animal should never be viewed as a complication or problem. Rather, validation of the human-animal bond can promote meaningful relationship-building between service providers and users.

Companion animals may not always have their own needs met, whether the owner is homeless or not. There are limited studies exploring the health of animals belonging to PEH specifically. Williams and Hogg [22] compared animals belonging to PEH with those of non-homeless owners and found no significant difference in terms of health and well-being. The three dogs seen in this study appeared in good health. Further studies investigating the health and well-being of animals of PEH are needed and may help guide future veterinary provision and support, as well as question some assumptions about what a good life for a dog looks like. This may even question some widely held tenets of ‘responsible pet ownership’.

The three service users ascribed significant benefits to dog ownership but there were also areas of concern: an inability/unwillingness to attend human health appointments because there was no one who could be trusted to look after the dog, problems with finding dog-inclusive accommodation, difficulties committing to employment. Contrasting effects on mental health were evident, ranging from positive emotional connections through to anxiety about losing their dog. A study by Merkouri et al [23] looked at benefits versus challenges of dog ownership and questioned a blanket view that dog ownership is invariably beneficial for human psychological health. Regardless of any definitive answer on this point (there probably isn’t one), the fact remains that PEH do have animals, and it is important that professionals acknowledge this choice and circumstance. The three service users interviewed in this study did not state any major healthcare access issues, which contrasted with some comments from service providers. This may be because the service users were all staying at a single location in Edinburgh, with facilities able to accommodate animals. Another factor might be that the three service users were relatively recently made homeless and therefore not in such need of health services: health progressively deteriorates the longer a person experiences homelessness.

Is joint healthcare a viable option? Joint healthcare is not a recognised term or medical discipline in the UK. In the projects represented through these participant narratives, there are clear examples of joint healthcare thinking and planning. These include inter-professional communication to facilitate individual care solutions for humans and their animals, and veterinary and human medical professionals providing combined outreach services, sometimes at the same facility. Transdisciplinarity, defined as the transcending of classical disciplinary boundaries to address complex problems and societal needs [24], is a hallmark of One Health for many proponents [25]. The context of addressing complex healthcare needs of PEH and companion animals does indeed call for transdisciplinary approaches. Coordinated teams consisting of human and veterinary staff, not just physicians but mental health and social support practitioners, and various animal health support workers, e.g., animal behaviourists, could hugely benefit these communities.

As mentioned in the introduction, in North America a small number of ‘One Health clinics’ provide combined veterinary, medical and social services to vulnerably-housed migrant populations [8], or people experiencing homelessness. Lem [7], Yang et al [9], Rock et al [26] and Bergia et al [10] describe outreach programmes which partner veterinarians with human health professionals. These examples of One Health clinics arose through the identification of gaps in human healthcare needs; by first meeting the animal healthcare needs, they created a safe environment, which encouraged human owners to engage with providers about their own healthcare needs. The concept of multi-species medicine feels new, even radical, but there are precedents. Schelling et al [27] described combined vaccination clinics for the children and livestock of pastoralist families in Chad: another example of a marginalised community where professionals can collaborate to provide multi-species healthcare. An interesting historical example was seen in the work of British diabetologist, Leslie Duncan (1922−51). Trained as both a veterinarian and a physician, he was a pioneer in the treatment of diabetes and for a time operated a multi-species outpatient clinic at The Royal Infirmary of Edinburgh [28]. These examples and others, though infrequent, can help us explore the ‘one-ness’ of One Health. These latter two examples focus on the resource sharing opportunities and health benefits posed through collaboration between human and animal healthcare service providers. The North American examples of One Health clinics serving marginalised communities are not limited to resource sharing but focus on creating impactful connections between service user and provider alongside those collaborations between different disciplines. Such connection and collaboration create long-lasting engagement with long term health benefits for humans and animals.

This paper is the first to discuss these ideas in the UK context. Questions remain as regards the need and scalability of the ideas presented here. Notably, the proportion of PEH with a companion animal in the UK remains unclear; more research is needed. In one study, 10% of PEH were reported as seeking animal-friendly housing [29]. PEH with an animal are therefore a minority within the homeless community as a whole, but their needs deserve to be met, especially as they are a group subject to multiple levels of exclusion and erasure.

Other recent examples of One Health clinics in North America quantify positive attributes of hosting joint clinics. Aguirre Siliezar et al [30] presented data indicating that the majority of respondents (>50% of animal owners) would prefer to go to a clinic where they could get services for themselves and their “pets” at the same time. Mitchell et al [12] describe the establishment of mobile One Health clinics in Pima County, Arizona, held in public parks, homeless shelters or libraries. Again, the 83% of studied clients who said they would return to the clinic in future is suggestive of a positive response. This is further borne out in a survey of attendees of the Seattle One Health Clinic, in which half of all clients returned to the clinic for a follow up visit, including those who initially had only sought animal health care services (Rejto et al [31]).

In contrast with the published examples from North America, this study found that the future for joint healthcare in the UK may not necessarily lie in the establishment of ‘one stop’ multi-species clinics. Perhaps it is lack of familiarity with the opportunities that such One Health clinics present, or perhaps differing systems of national healthcare provision, both public and private, play a role here.

There is obvious resource-saving in having a single space from which animal and human health professionals can operate, but our study shows that co-location risks failure if trust and relationship-building are not prioritised first. For the current UK context, a better term than joint healthcare might be ‘coordinated healthcare,’ delivered through improved awareness, communication and collaboration across disciplines. Such coordination demonstrates the application of the foundational principles of One Health. Translating these principles into practice is not straightforward: there is a need to adapt them to the particularities of the ‘urban ecosystem’ and healthcare professionals must learn to meet people and their companion animals where they are, to listen and to attune [32] and, in doing so, develop equitable partnering approaches. In the UK, coordinated healthcare need not rely on a single location from which services can be delivered. Nor is coordinated healthcare about vets functioning as human medics and vice versa. It is about being aware of complementary professional contributions and how to develop effective partnerships. In relation to service users, it is about shifting from asking, ‘What’s wrong with you?’ to asking ‘What happened to you?’ [33]. This represents a paradigmatic shift in incorporating trauma-informed practice into healthcare delivery. It is also about remembering that the animal that is often present is equally deserving of attention.

The third sector props up homelessness services in the UK, but this is a precarious and inconsistent form of support. Such volatility risks alienating wary, traumatised service users who, living lives on the periphery of mainstream society, are already used to being disappointed when services suddenly disappear. Good evidence on the ways in which UK-based public, private and third sector organisations can better integrate is needed to help advocate for support from local and national decision-makers. The existing examples of coordinated provision of healthcare in North America typically have undergraduate education at their core, often using a philosophy of ‘service learning’ through student-led clinics. Whilst this educational opportunity is essential to ensure that the next generation of healthcare professionals is trauma-informed and One Health savvy, it should not be the sole solution to service provision.

To develop more permanent and sustainable networks, raised awareness through Continuing Professional Development (CPD) and networking are critical. This type of work necessitates community of practice approaches [34,35]. Within the UK veterinary and medical professions, there is considerable interest in what is becoming known as ‘contextualised care’ (sometimes referred to as ‘spectrum of care’ or ‘pragmatic care’). Contextualised care refers to options that are developed collaboratively with the patient, taking stock of research evidence, patient preferences, clinical presentation, economics (especially in veterinary medicine) and situational context [36,37]. The treatment of animals belonging to PEH is a prime example of contextualised care due to the specific and challenging contexts encountered. Knowledge of core One Health principles is variable amongst UK healthcare professionals and there are ongoing debates about how these should inform practice. Using communities of practice to increase awareness of practical One Health applications through coordinated care will multiply the numbers of people exploring and utilising these approaches. Groups of professionals working together to provide contextualised care for their human and non-human patients is a One Health vision worthy of pursuit and may, in time, result in dedicated One Health clinics that develop similarities with their North America counterparts. Indeed, one of the study participants here represented a grassroots charity who provide animal companionship services to vulnerable adults. Such services incorporate liaising with human and animal health professionals to provide holistic care that attempts to maintain the human animal bond between owners and their animal companions.

This study has limitations. Service providers and users from only a few locations in the UK were included. The small number of service users from a single location presents the major limitation. A proposed future direction would be a UK-wide study, using another methodology suitable for larger sample sizes, that includes more service users as well as representatives from the multiple public, private and third sector organisations that support healthcare for PEH. A quantitative or mixed methods study would complement the qualitative approach used here. This study had a disproportionate number of service providers from Edinburgh, and a future study should have a more balanced geographical distribution of both service users and providers. Finally, whilst the majority of PEH with a companion animal have a dog as their companion animal, as did the three service users in this study, service providers and the literature also describe service users with cats, rodents and other species. Therefore, any future study should include PEH with greater species diversity, to facilitate a broader understanding of the animal healthcare needs within this population.
