# Supplementary material for: ‘My dog is my life’: One Health approaches to healthcare for people and their companion animals experiencing homelessness in the United Kingdom
Source: PLoS One. 2026 Sep 3;21(9):e0354517. doi: 10.1371/journal.pone.0354517 (PMC13541128; doi:10.1371/journal.pone.0354517)
Supplement: Supplementary file 1 [file pone.0354517.s001.DOC]

Becky response to reviewers

Dear Sir/Madam,

We thank you for your consideration of our paper for publication for PlosOne. We have reviewed the manuscript in line with comments made by both reviewers and offer a revised manuscript. Substantial changes have been highlighted in yellow. We also provide a clean draft that has been further proof edited.

For simplicity we have provided an explanation for our responses in the order in which the comments were made as below:

When submitting your revision, we need you to address these additional requirements.

1. Please ensure that your manuscript meets PLOS ONE's style requirements, including those for file naming. The PLOS ONE style templates can be found at

[https://journals.plos.org/plosone/s/file?id=wjVg/PLOSOne_formatting_sample_main_body.pdf](https://eur02.safelinks.protection.outlook.com/?url=https%3A%2F%2Fjournals.plos.org%2Fplosone%2Fs%2Ffile%3Fid%3DwjVg%2FPLOSOne_formatting_sample_main_body.pdf&data=05|02||5a9f4bb47eb049844f0108de4c5297f0|2e9f06b016694589878910a06934dc61|0|0|639032114776204418|Unknown|TWFpbGZsb3d8eyJFbXB0eU1hcGkiOnRydWUsIlYiOiIwLjAuMDAwMCIsIlAiOiJXaW4zMiIsIkFOIjoiTWFpbCIsIldUIjoyfQ%3D%3D|0|||&sdata=laSG%2FBC1aBtOeItUJvnHhez%2FKF9BuWJMVcKWvzTcXjs%3D&reserved=0) and

[https://journals.plos.org/plosone/s/file?id=ba62/PLOSOne_formatting_sample_title_authors_affiliations.pdf](https://eur02.safelinks.protection.outlook.com/?url=https%3A%2F%2Fjournals.plos.org%2Fplosone%2Fs%2Ffile%3Fid%3Dba62%2FPLOSOne_formatting_sample_title_authors_affiliations.pdf&data=05|02||5a9f4bb47eb049844f0108de4c5297f0|2e9f06b016694589878910a06934dc61|0|0|639032114776226344|Unknown|TWFpbGZsb3d8eyJFbXB0eU1hcGkiOnRydWUsIlYiOiIwLjAuMDAwMCIsIlAiOiJXaW4zMiIsIkFOIjoiTWFpbCIsIldUIjoyfQ%3D%3D|0|||&sdata=Gl%2FPr0KqcNpvfw4Otn3KKzdxG2428M6Ns88Cc%2Bv8S5g%3D&reserved=0)

We have revised the manuscript to take into account your style requirements. We trust we have not overlooked anything.

2. In the online submission form you indicate that your data is not available for proprietary reasons and have provided a contact point for accessing this data. Please note that your current contact point is a co-author on this manuscript. According to our Data Policy, the contact point must not be an author on the manuscript and must be an institutional contact, ideally not an individual. Please revise your data statement to a non-author institutional point of contact, such as a data access or ethics committee, and send this to us via return email. Please also include contact information for the third party organization, and please include the full citation of where the data can be found.

It is unclear where this should appear in the manuscript. The corresponding author is an institutional contact and was the guarantor for the data. It would not make sense to provide you with the email for the ethics committee as they are independent of the data held. There is no full citation to provide you with.

If the reviewer comments include a recommendation to cite specific previously published works, please review and evaluate these publications to determine whether they are relevant and should be cited. There is no requirement to cite these works unless the editor has indicated otherwise.

We found the extra references supplied by the reviewers to be excellent resources and are grateful to have been signposted to them. These publications have been included in the revised manuscript, with the reference list and numbering in the main text updated accordingly.

**Reviewers' comments:**

Reviewer's Responses to Questions.

**Comments to the Author** 
 
1. Is the manuscript technically sound, and do the data support the conclusions? 
 
The manuscript must describe a technically sound piece of scientific research with data that supports the conclusions. Experiments must have been conducted rigorously, with appropriate controls, replication, and sample sizes. The conclusions must be drawn appropriately based on the data presented.

Reviewer #1: Yes

Reviewer #2: Partly.

2. Has the statistical analysis been performed appropriately and rigorously?

Reviewer #1: I Don't Know We believe this to be N/A, as per reviewer 2, as there was no statistical analysis as part of this study.

Reviewer #2: N/A

3. Have the authors made all data underlying the findings in their manuscript fully available? 
 
The [PLOS Data policy](https://eur02.safelinks.protection.outlook.com/?url=http%3A%2F%2Ftrack.editorialmanager.com%2FCL0%2Fhttp%3A%252F%252Fwww.plosone.org%252Fstatic%252Fpolicies.action%2523sharing%2F1%2F010f019b8d1d9a1b-aa6e16fe-2213-4933-90c2-9fc533dc4737-000000%2F0XMLS-QHQDdrdCglCetNJZ9LmfHgsMPIVHZgP7udPBw%3D244&data=05|02||5a9f4bb47eb049844f0108de4c5297f0|2e9f06b016694589878910a06934dc61|0|0|639032114776250677|Unknown|TWFpbGZsb3d8eyJFbXB0eU1hcGkiOnRydWUsIlYiOiIwLjAuMDAwMCIsIlAiOiJXaW4zMiIsIkFOIjoiTWFpbCIsIldUIjoyfQ%3D%3D|0|||&sdata=0WkBIHbGYSBeWmH8J0AlNWkVN5Rr0JIuM3uGp3TxBtw%3D&reserved=0) requires authors to make all data underlying the findings described in their manuscript fully available without restriction, with rare exception (please refer to the Data Availability Statement in the manuscript PDF file). The data should be provided as part of the manuscript or its supporting information, or deposited to a public repository. For example, in addition to summary statistics, the data points behind means, medians and variance measures should be available. If there are restrictions on publicly sharing data—e.g. participant privacy or use of data from a third party—those must be specified.

Reviewer #1: No

Reviewer #2: No

In line with qualitative research norms and as discussed previously, with the Journal, there is limited data availability. This has been clearly explained in the manuscript.

4. Is the manuscript presented in an intelligible fashion and written in standard English? 
 
PLOS ONE does not copyedit accepted manuscripts, so the language in submitted articles must be clear, correct, and unambiguous. Any typographical or grammatical errors should be corrected at revision, so please note any specific errors here.

Reviewer #1: Yes

Reviewer #2: Yes

5. Review Comments to the Author 
 
Please use the space provided to explain your answers to the questions above. You may also include additional comments for the author, including concerns about dual publication, research ethics, or publication ethics. (Please upload your review as an attachment if it exceeds 20,000 characters)

Reviewer #1: The manuscript titled ‘One Health approaches to healthcare for people and their companion animals experiencing homelessness’ contributes to science by providing rich qualitative data that applies the One Health Framework to assess themes related to healthcare needs and challenges and the feasibility of joint healthcare. 
The results of this study support the authors' claims that the implementation of consistent and sustainable joint healthcare could be a feasible method to increase human and veterinary healthcare utilization among people facing homelessness with their dogs. The data is also in alignment with the authors’ interpretation that building trust, stigmatization, role of the human-animal bond, consistency of services and providers, housing, and trauma must also be addressed to implement sustainable joint healthcare services for houseless population with animals. 
This paper can be improved by contextualizing the study and its findings within the current literature on one health clinics in North America. The references do not include several articles on integrated or dual healthcare among populations experiencing homelessness that have been published in the last five years. The introduction could be stronger by providing a summary of the state of the science. The Discussion could be strengthened by addressing how these findings build on or differ from current literature. 
The authors appropriately describe restrictions associated with data availability and provide a sound rationale. I recommend the authors provide further information surrounding the inclusion and exclusion criteria applied to participant recruitment to support reproducibility. The limitations could be strengthened by including the implications of the small sample size of service providers and study sites. 
I highly encourage the authors to integrate and contextualize the current literature in the manuscript and resubmit a revised version.

Thank you for the encouraging and reflective review. We have added additional detail in the introduction, approach and discussion sections, as well as incorporating the offered references by reviewer 2 below. The inclusion and exclusion criteria have been further elucidated.

Reviewer #2: Introduction 
1. The introduction section provides only minimal background information and context. Importantly, there is a lack of awareness or mention of current One Health models in practice, see for example: 
1. Rejto N, Ramirez V, Fenelon HT, Xie M, Kuehl K, Terry A, Tin AH, Tabor E, Schneier K, Nee A, Richer A, Rabinowitz P, Meisner J. The One Health Clinic: Care for Young Adults and Companion Animals Experiencing Homelessness. J Prim Care Community Health. 2025 Jan-Dec;16: 
2. Aguirre Siliezar K, Patel S, Chande R, Joiner A, Hoover MC, Mathis MW, Hendrickson J, Siliezar J, Jankowski K. Utilizing the Human Animal Bond to Promote Preventive Care Engagement in Underserved Communities: A Descriptive Study of 2 U.S. One Health Clinics. J Prim Care Community Health. 2025 Jan-Dec;16 
3. Wagner K, Singletary M, Kohler LN, Ellingson KD, Wagner J, Pogreba-Brown K, Cullen TA. Integrating Human-Animal Care Through a Public Health-Driven One Health Clinic Model in Pima County, Arizona, October 2023-February 2024. Public Health Rep. 2026 Jan-Feb;141(1):31-37. 
4. Tin AH, Kuehl K, Terry A, Meisner J, Ramirez V, Tabor E, Rabinowitz P. A One Health Clinic for People Experiencing Homelessness and Their Animals: Treating the Human-Animal Unit. Ann Fam Med. 2022 Sep-Oct;20(5):487.

Thank you for these references, which have been incorporated.

2. In. addition, there appear to be sone random open parenthesis on the last line of the first paragraph We could not locate these

RESEARCH APPROACH 
Participant Recruitment 
1. I would have liked better details about participant recruitment “#2) Identifying service users who would not have their mental health compromised through participation.” How? My whom? All researchers were veterinarians. We have added a sentence to confirm that the identification was done by the hostel staff, who have extensive experience in working with this population.
2. Snowball sampling through initial research team contacts: Were the Service Users also through previous contacts of research team? Or through the shelter (which wouldn’t be their contacts)? We have clarified this in the manuscript, and refer back to the point above.

3. Tables 1 and 2 could be combined- duplicative 
4. Table 3 is too identifiable, sensitive gender information, and the small sample size, even with peudonmyms (is that necessary?) would delete or combine with tables 1 and 2. We thank you for these comments and have given this matter careful consideration. However, we feel that combining the two tables would complicate the data presentation. Furthermore, the use of pseudonyms and number of other people found in each location is, we feel, sufficient to maintain anonymity.

5. Table 1: The term “Veterinary Nurse” is not used in the US. Is that a Veterinary Tech? It would help US readers understand if it was defined, We have added “akin to vet tech” in main manuscript for clarification.

6. Table 2: The abstract seems to focus on dogs, but Table 2 inclusion criteria for service users says “companion animals.” All respondents had dogs. Was that happenstance or did they actually select for dogs. If so, inclusion criteria should be “with dogs.” If not, what if some participants had had cats? Suggest that the authors need to clarify whether other animals were present or did selection criteria exclude them. Thank you. We have clarified that the three participants selected happened to have dogs, whilst in the discussion, noted that although dogs are the most common species found as companion animals to PEH in the UK, other species can also be present.

7. Table 3: The small sample size and selection criteria needs to be considered here- are qualitative data from 1 Trans person and 2 Males based out of one shelter representative of a larger population? It would appear unlikely. We have addressed this in the study limitations.

Ethics and Consent 
1. Were any incentives offered to participants for their time and participation in the study? Since dealing with a vulnerable population this should be considered. As stated in the original manuscript and highlighted for clarity -no incentives were offered.

Participant Interviewing 
1. Is there bias in response because research RS “provide veterinary support to animal and their humans experiencing homelessness in the UK.” (See “Researcher positionalities”) If the homelessness population is small and many know each other (noted later), will a veterinarian providing care to this community get unbiased responses from service users? RS is a volunteer providing veterinary care in the southwest, the three service users were all in Edinburgh. RS did not volunteer for the duration of this study.

Data Processing and analysis: 
1. I am having a hard time with their qualitative methodology due to the small sample size. This reviwer questions whether IPA is an appropriate analytic method given the sample size, and what they are trying to force into that box. They write: 
 
“Interpretative Phenomenological Analysis (IPA) was selected for data analysis as best 
suited for exploring peoples’ lived experiences of a specific phenomenon [10]. IPA’s 
double hermeneutic approach, in which participants provide their own interpretation of 
their experience which the researcher in turn interprets, is considered appropriate for 
‘topics which are complex, ambiguous and emotionally laden’ [11]. The number of 
participants in this study (n=13) exceeded that typically used for an IPA project (usually 
<10). Small sample sizes are desirable to enable the detailed idiographic analysis of 
lived experience using IPA. However larger sample sizes (as here) avoid the risk of loss 
of detail when interviews are tightly focused around a central research question or 
when individuals share certain important characteristics, in this case the common 
experience of homelessness [10].” 
 
• There are only 3 PEH interviews, 2 Males and 1 Tran person, all with dogs, coming from one shelter. Again, this seems unlikely to be full representative of the greater population in the 6 UK locations identified (page 4)

This is acknowledged in the study limitations: The number of service users was small, however this is a benefit for IPA based studies, with Smith el al (2022) recommending n=3-10. Participants are selected to be representative of a perspective, rather than a population.  This study was a multi-perspective study, comparing and contrasting the IPA of service providers and service users, in order to gain a broad and holistic understanding of the healthcare needs and challenges of the specific population studied here.

• There are 13 respondents, but only 3 PEH respondents. The authors state that their respondent size is a positive to “avoid the risk of loss of detail” but they are lumping the 3 PEH into the group of healthcare providers, and also healthcare providers with housing services and animal welfare. This could potentially dilute the findings for any particular subgroup.

See above -the two groups are not lumped together, they are treated as two linked groups, linked through the phenomenon of homelessness. Multi-perspective studies are a recognised use of IPA, in order to gain “ a multi-faceted account of the phenomenon” (Smith et al 2022).

• I think this is an informative study, but I don’t know that IPA makes sense.

We appreciate the question but disagree as IPA has been proven an excellent choice of methodology for providing a qualitative study of complex emotional subjects within a small sample cohort. • 
 
Results 
Research Question 1: 
Healthcare Needs 
1. There is reference to Spay/Neuter challenges, but this is not referenced again in the text. Suggest either expanding or deleting. This has been deleted.

2. Return to later Sally (GP)’s quote….are there community vets who say the same thing? I mean, it’s the case in housed families too. I’m not sure what this refers to?

3. Is the ransom piece necessary? Will a joint clinic influence this? Why is this under “Animal healthcare”?. This statement has been deleted.

4. Page 13, section iii : “According to service providers, every PEH they encountered also had substance abuse concerns related to previous trauma.” To state that every had this concern appears to be an exaggeration, and appears pejorative. This was the statement of every single service provider and so not pejorative with respect to being an accurate reflection of their interview statements. Whether this is truly representative of every service user cannot be determined in this study. We have extended the text in this section to qualify the statement further.

Healthcare Challenges 
1. Section i : I assume the journal will advise on quote punctuation, but I find their swap of single and double quotation marks confusing

We are using the convention of single quotes as the first layer of quotation marks, with quotes-within-quotes being allocated double quotation marks. This is in line with current style guides, e.g. Hart’s Rules, and is the convention most used in contemporary publishing. Occasionally in some publications, the regime is reversed, i.e. double quotes is the first layer and quotes-within-quotes is single quotation marks. We found no explicit guidelines on this so used the commonest variant but are happy for the publisher to alter if they have a preference.

2. Page 15, section ii : This is hard to interpret just from the writing : “Chris feared medical professionals insisting his dog was removed or otherwise losing his dog while he was attending an appointment (e.g. if stolen while tied up outside a clinic):” Would clarify Not quite sure what this is questioning?
 
 
DISCUSSION 
1. Page 23, paragraph starting with “Our service users…”, end of paragraph states some limitations of their 3 people. Have now started paragraph
a. Are there more service providers in Edinburgh vs the 5 other locations? No, just more identified there due to snowballing. Acknowledged this in study limitations.
b. Gender differences in need? Not quite sure what the reviewer is asking here -are they querying what we thought about this? The sample size is too small to make any statement with reference to gender. Can add something to that effect?

2. Page 24, paragraph starting with “In North America”… see above- recommend they acknowledge experience of clinics eslsewhere, particularly because some of their findings are at odds with what others have reported.regarding the acceptability of the integrated care model. Thank you, we have added this contextual information and comparison with our UK experience to the discussion

3. Page 26, first full paragraph starting with “This study has some limitations” – Again, they are claiming there N for IPA is high, but they are combining two EXCEEDINGLY disparate groups, and their number of service users is very very small. Study lacks gender diversity, species diversity, a description of location diversity (e.g., services available in one location vs another . The two groups are not exceedingly disparate -the groups are linked through the connection with homelessness. Location and species diversity are acknowledged in the limitations.
