# Supplementary material for: ‘My dog is my life’: One Health approaches to healthcare for people and their companion animals experiencing homelessness in the United Kingdom
Source: PLoS One. 2026 Sep 3;21(9):e0354517. doi: 10.1371/journal.pone.0354517 (PMC13541128; doi:10.1371/journal.pone.0354517)
Supplement: Peer review history

# Peer review: Decision Letter 0

**DOI:** 10.1371/journal.pone.0354517.r001

**Authors:** Ulrike Gertrud Munderloh

5 Jan 2026

Dear Dr. Cousquer,

Please submit your revised manuscript by Feb 19 2026 11:59PM. If you will need more time than this to complete your revisions, please reply to this message or contact the journal office at plosone@plos.org

- A letter that responds to each point raised by the academic editor and reviewer(s). You should upload this letter as a separate file labeled 'Response to Reviewers'.
- A marked-up copy of your manuscript that highlights changes made to the original version. You should upload this as a separate file labeled 'Revised Manuscript with Track Changes'.
- An unmarked version of your revised paper without tracked changes. You should upload this as a separate file labeled 'Manuscript'.

If you would like to make changes to your financial disclosure, please include your updated statement in your cover letter. Guidelines for resubmitting your figure files are available below the reviewer comments at the end of this letter.

If applicable, we recommend that you deposit your laboratory protocols in protocols.io to enhance the reproducibility of your results. Protocols.io assigns your protocol its own identifier (DOI) so that it can be cited independently in the future. For instructions see: https://journals.plos.org/plosone/s/submission-guidelines#loc-laboratory-protocols. Additionally, PLOS ONE offers an option for publishing peer-reviewed Lab Protocol articles, which describe protocols hosted on protocols.io. Read more information on sharing protocols at https://plos.org/protocols?utm_medium=editorial-email&utm_source=authorletters&utm_campaign=protocols.

We look forward to receiving your revised manuscript.

Kind regards,

Ulrike Gertrud Munderloh, Ph.D.

Academic Editor

PLOS One

Journal requirements:

When submitting your revision, we need you to address these additional requirements.

1. Please ensure that your manuscript meets PLOS ONE's style requirements, including those for file naming. The PLOS ONE style templates can be found at

https://journals.plos.org/plosone/s/file?id=wjVg/PLOSOne_formatting_sample_main_body.pdf and

https://journals.plos.org/plosone/s/file?id=ba62/PLOSOne_formatting_sample_title_authors_affiliations.pdf

2. In the online submission form you indicate that your data is not available for proprietary reasons and have provided a contact point for accessing this data. Please note that your current contact point is a co-author on this manuscript. According to our Data Policy, the contact point must not be an author on the manuscript and must be an institutional contact, ideally not an individual. Please revise your data statement to a non-author institutional point of contact, such as a data access or ethics committee, and send this to us via return email. Please also include contact information for the third party organization, and please include the full citation of where the data can be found.

If the reviewer comments include a recommendation to cite specific previously published works, please review and evaluate these publications to determine whether they are relevant and should be cited. There is no requirement to cite these works unless the editor has indicated otherwise.

[Note: HTML markup is below. Please do not edit.]

Reviewers' comments:

Reviewer's Responses to Questions

**Comments to the Author**

1. Is the manuscript technically sound, and do the data support the conclusions?

Reviewer #1: Yes

Reviewer #2: Partly

**********

2. Has the statistical analysis been performed appropriately and rigorously?

Reviewer #1: I Don't Know

Reviewer #2: N/A

**********

3. Have the authors made all data underlying the findings in their manuscript fully available?

The PLOS Data policy

Reviewer #1: No

Reviewer #2: No

**********

4. Is the manuscript presented in an intelligible fashion and written in standard English?

Reviewer #1: Yes

Reviewer #2: Yes

**********

Reviewer #1: The manuscript titled ‘One Health approaches to healthcare for people and their companion animals experiencing homelessness’ contributes to science by providing rich qualitative data that applies the One Health Framework to assess themes related to healthcare needs and challenges and the feasibility of joint healthcare.

The results of this study support the authors' claims that the implementation of consistent and sustainable joint healthcare could be a feasible method to increase human and veterinary healthcare utilization among people facing homelessness with their dogs. The data is also in alignment with the authors’ interpretation that building trust, stigmatization, role of the human-animal bond, consistency of services and providers, housing, and trauma must also be addressed to implement sustainable joint healthcare services for houseless population with animals.

This paper can be improved by contextualizing the study and its findings within the current literature on one health clinics in North America. The references do not include several articles on integrated or dual healthcare among populations experiencing homelessness that have been published in the last five years. The introduction could be stronger by providing a summary of the state of the science. The Discussion could be strengthened by addressing how these findings build on or differ from current literature.

The authors appropriately describe restrictions associated with data availability and provide a sound rationale. I recommend the authors provide further information surrounding the inclusion and exclusion criteria applied to participant recruitment to support reproducibility. The limitations could be strengthened by including the implications of the small sample size of service providers and study sites.

I highly encourage the authors to integrate and contextualize the current literature in the manuscript and resubmit a revised version.

Reviewer #2: Introduction

1. The introduction section provides only minimal background information and context. Importantly, there is a lack of awareness or mention of current One Health models in practice, see for example:

1. Rejto N, Ramirez V, Fenelon HT, Xie M, Kuehl K, Terry A, Tin AH, Tabor E, Schneier K, Nee A, Richer A, Rabinowitz P, Meisner J. The One Health Clinic: Care for Young Adults and Companion Animals Experiencing Homelessness. J Prim Care Community Health. 2025 Jan-Dec;16:

2. Aguirre Siliezar K, Patel S, Chande R, Joiner A, Hoover MC, Mathis MW, Hendrickson J, Siliezar J, Jankowski K. Utilizing the Human Animal Bond to Promote Preventive Care Engagement in Underserved Communities: A Descriptive Study of 2 U.S. One Health Clinics. J Prim Care Community Health. 2025 Jan-Dec;16

3. Wagner K, Singletary M, Kohler LN, Ellingson KD, Wagner J, Pogreba-Brown K, Cullen TA. Integrating Human-Animal Care Through a Public Health-Driven One Health Clinic Model in Pima County, Arizona, October 2023-February 2024. Public Health Rep. 2026 Jan-Feb;141(1):31-37.

4. Tin AH, Kuehl K, Terry A, Meisner J, Ramirez V, Tabor E, Rabinowitz P. A One Health Clinic for People Experiencing Homelessness and Their Animals: Treating the Human-Animal Unit. Ann Fam Med. 2022 Sep-Oct;20(5):487.

2. In. addition, there appear to be sone random open parenthesis on the last line of the first paragraph

RESEARCH APPROACH

Participant Recruitment

1. I would have liked better details about participant recruitment “#2) Identifying service users who would not have their mental health compromised through participation.” How? My whom? All researchers were veterinarians.

2. Snowball sampling through initial research team contacts: Were the Service Users also through previous contacts of research team? Or through the shelter (which wouldn’t be their contacts)?

3. Tables 1 and 2 could be combined- duplicative

4. Table 3 is too identifiable, sensitive gender information, and the small sample size, even with peudonmyms (is that necessary?) would delete or combine with tables 1 and 2.

5. Table 1: The term “Veterinary Nurse” is not used in the US. Is that a Veterinary Tech? It would help US readers understand if it was defined,

6. Table 2: The abstract seems to focus on dogs, but Table 2 inclusion criteria for service users says “companion animals.” All respondents had dogs. Was that happenstance or did they actually select for dogs. If so, inclusion criteria should be “with dogs.” If not, what if some participants had had cats? Suggest that the authors need to clarify whether other animals were present or did selection criteria exclude them.

7. Table 3: The small sample size and selection criteria needs to be considered here- are qualitative data from 1 Trans person and 2 Males based out of one shelter representative of a larger population? It would appear unlikely.

Ethics and Consent

1. Were any incentives offered to participants for their time and participation in the study? Since dealing with a vulnerable population this should be considered.

Participant Interviewing

1. Is there bias in response because research RS “provide veterinary support to animal and their humans experiencing homelessness in the UK.” (See “Researcher positionalities”) If the homelessness population is small and many know each other (noted later), will a veterinarian providing care to this community get unbiased responses from service users?

Data Processing and analysis:

1. I am having a hard time with their qualitative methodology due to the small sample size. This reviwer questions whether IPA is an appropriate analytic method given the sample size, and what they are trying to force into that box. They write:

“Interpretative Phenomenological Analysis (IPA) was selected for data analysis as best

suited for exploring peoples’ lived experiences of a specific phenomenon [10]. IPA’s

double hermeneutic approach, in which participants provide their own interpretation of

their experience which the researcher in turn interprets, is considered appropriate for

‘topics which are complex, ambiguous and emotionally laden’ [11]. The number of

participants in this study (n=13) exceeded that typically used for an IPA project (usually

<10). Small sample sizes are desirable to enable the detailed idiographic analysis of

lived experience using IPA. However larger sample sizes (as here) avoid the risk of loss

of detail when interviews are tightly focused around a central research question or

when individuals share certain important characteristics, in this case the common

experience of homelessness [10].”

• There are only 3 PEH interviews, 2 Males and 1 Tran person, all with dogs, coming from one shelter. Again, this seems unlikely to be full representative of the greater population in the 6 UK locations identified (page 4)

• There are 13 respondents, but only 3 PEH respondents. The authors state that their respondent size is a positive to “avoid the risk of loss of detail” but they are lumping the 3 PEH into the group of healthcare providers, and also healthcare providers with housing services and animal welfare. This could potentially dilute the findings for any particular subgroup.

• I think this is an informative study, but I don’t know that IPA makes sense.

• Results

Research Question 1:

Healthcare Needs

1. There is reference to Spay/Neuter challenges, but this is not referenced again in the text.Suggest either expanding or deleting.

2. Return to later Sally (GP)’s quote….are there community vets who say the same thing? I mean, it’s the case in housed families too.

3. Is the ransom piece necessary? Will a joint clinic influence this? Why is this under “Animal healthcare”?.

4. Page 13, section iii : “According to service providers, every PEH they encountered also had substance abuse concerns related to previous trauma.” To state that every had this concern appears to be an exaggeration, and appears pejorative.

Healthcare Challenges

1. Section i : I assume the journal will advise on quote punctuation, but I find their swap of single and double quotation marks confusing

2. Page 15, section ii : This is hard to interpret just from the writing : “Chris feared medical professionals insisting his dog was removed or otherwise losing his dog while he was attending an appointment (e.g. if stolen while tied up outside a clinic):” Would clarify

DISCUSSION

1. Page 23, paragraph starting with “Our service users…”, end of paragraph states some limitations of their 3 people.

a. Are there more service providers in Edinburgh vs the 5 other locations?

b. Gender differences in need?

2. Page 24, paragraph starting with “In North America”… see above- recommend they acknowledge experience of clinics eslsewhere, particularly because some of their findings are at odds with what others have reported.regarding the acceptability of the integrated care model.

3. Page 26, first full paragraph starting with “This study has some limitations” – Again, they are claiming there N for IPA is high, but they are combining two EXCEEDINGLY disparate groups, and their number of service users is very very small. Study lacks gender diversity, species diversity, a description of location diversity (e.g., services available in one location vs another

**********

what does this mean?). If published, this will include your full peer review and any attached files.

If you choose “no”, your identity will remain anonymous but your review may still be made public.

**Do you want your identity to be public for this peer review?**  For information about this choice, including consent withdrawal, please see our Privacy Policy

Reviewer #1: **Yes:**  Natalie Rejto

Reviewer #2: No

**********

[NOTE: If reviewer comments were submitted as an attachment file, they will be attached to this email and accessible via the submission site. Please log into your account, locate the manuscript record, and check for the action link "View Attachments". If this link does not appear, there are no attachment files.]

To ensure your figures meet our technical requirements, please review our figure guidelines: https://journals.plos.org/plosone/s/figures

You may also use PLOS’s free figure tool, NAAS, to help you prepare publication quality figures: https://journals.plos.org/plosone/s/figures#loc-tools-for-figure-preparation.

NAAS will assess whether your figures meet our technical requirements by comparing each figure against our figure specifications.

# Author comment: Author response to Decision Letter 1

**DOI:** 10.1371/journal.pone.0354517.r002

20 Feb 2026

Dear Editors and Reviewers

Many thanks for your time and attention in reviewing this work. We are grateful for the feedback received. In this response, we have included both a clean manuscript (dated 18.02.26) and a manuscript with the changes tracked. The latter won't show the formatting changes I made to the clean manuscript in accordance with your style guidelines.

I am copying the response to the decision letter below and have also uploaded this as a separate document (where all our answers are in red, making them easier to identify). I trust this is easy to follow.

Yours Sincerely

Dr Glen Cousquer.

Dear Sir/Madam,

We thank you for your consideration of our paper for publication for PlosOne. We have reviewed the manuscript in line with comments made by both reviewers and offer a revised manuscript. Substantial changes have been highlighted in yellow. We also provide a clean draft that has been further proof edited.

For simplicity we have provided an explanation for our responses in the order in which the comments were made as below:

When submitting your revision, we need you to address these additional requirements.

1. Please ensure that your manuscript meets PLOS ONE's style requirements, including those for file naming. The PLOS ONE style templates can be found at

https://journals.plos.org/plosone/s/file?id=wjVg/PLOSOne_formatting_sample_main_body.pdf and

https://journals.plos.org/plosone/s/file?id=ba62/PLOSOne_formatting_sample_title_authors_affiliations.pdf

We have revised the manuscript to take into account your style requirements. We trust we have not overlooked anything.

2. In the online submission form you indicate that your data is not available for proprietary reasons and have provided a contact point for accessing this data. Please note that your current contact point is a co-author on this manuscript. According to our Data Policy, the contact point must not be an author on the manuscript and must be an institutional contact, ideally not an individual. Please revise your data statement to a non-author institutional point of contact, such as a data access or ethics committee, and send this to us via return email. Please also include contact information for the third party organization, and please include the full citation of where the data can be found.

It is unclear where this should appear in the manuscript. The corresponding author is an institutional contact and was the guarantor for the data. It would not make sense to provide you with the email for the ethics committee as they are independent of the data held. There is no full citation to provide you with.

If the reviewer comments include a recommendation to cite specific previously published works, please review and evaluate these publications to determine whether they are relevant and should be cited. There is no requirement to cite these works unless the editor has indicated otherwise.

We found the extra references supplied by the reviewers to be excellent resources and are grateful to have been signposted to them. These publications have been included in the revised manuscript, with the reference list and numbering in the main text updated accordingly.

Reviewers' comments:

Reviewer's Responses to Questions.

Comments to the Author

1. Is the manuscript technically sound, and do the data support the conclusions?

The manuscript must describe a technically sound piece of scientific research with data that supports the conclusions. Experiments must have been conducted rigorously, with appropriate controls, replication, and sample sizes. The conclusions must be drawn appropriately based on the data presented.

Reviewer #1: Yes

Reviewer #2: Partly.

2. Has the statistical analysis been performed appropriately and rigorously?

Reviewer #1: I Don't Know.

We believe this to be N/A, as per reviewer 2, as there was no statistical analysis as part of this study.

Reviewer #2: N/A

3. Have the authors made all data underlying the findings in their manuscript fully available?

The PLOS Data policy requires authors to make all data underlying the findings described in their manuscript fully available without restriction, with rare exception (please refer to the Data Availability Statement in the manuscript PDF file). The data should be provided as part of the manuscript or its supporting information, or deposited to a public repository. For example, in addition to summary statistics, the data points behind means, medians and variance measures should be available. If there are restrictions on publicly sharing data—e.g. participant privacy or use of data from a third party—those must be specified.

Reviewer #1: No

Reviewer #2: No

In line with qualitative research norms and as discussed previously, with the Journal, there is limited data availability. This has been clearly explained in the manuscript.

4. Is the manuscript presented in an intelligible fashion and written in standard English?

PLOS ONE does not copyedit accepted manuscripts, so the language in submitted articles must be clear, correct, and unambiguous. Any typographical or grammatical errors should be corrected at revision, so please note any specific errors here.

Reviewer #1: Yes

Reviewer #2: Yes

5. Review Comments to the Author

Please use the space provided to explain your answers to the questions above. You may also include additional comments for the author, including concerns about dual publication, research ethics, or publication ethics. (Please upload your review as an attachment if it exceeds 20,000 characters)

Reviewer #1: The manuscript titled ‘One Health approaches to healthcare for people and their companion animals experiencing homelessness’ contributes to science by providing rich qualitative data that applies the One Health Framework to assess themes related to healthcare needs and challenges and the feasibility of joint healthcare.

The results of this study support the authors' claims that the implementation of consistent and sustainable joint healthcare could be a feasible method to increase human and veterinary healthcare utilization among people facing homelessness with their dogs. The data is also in alignment with the authors’ interpretation that building trust, stigmatization, role of the human-animal bond, consistency of services and providers, housing, and trauma must also be addressed to implement sustainable joint healthcare services for houseless population with animals.

This paper can be improved by contextualizing the study and its findings within the current literature on one health clinics in North America. The references do not include several articles on integrated or dual healthcare among populations experiencing homelessness that have been published in the last five years. The introduction could be stronger by providing a summary of the state of the science. The Discussion could be strengthened by addressing how these findings build on or differ from current literature.

The authors appropriately describe restrictions associated with data availability and provide a sound rationale. I recommend the authors provide further information surrounding the inclusion and exclusion criteria applied to participant recruitment to support reproducibility. The limitations could be strengthened by including the implications of the small sample size of service providers and study sites.

I highly encourage the authors to integrate and contextualize the current literature in the manuscript and resubmit a revised version.

Thank you for the encouraging and reflective review. We have added additional detail in the introduction, approach and discussion sections, as well as incorporating the offered references by reviewer 2 below. The inclusion and exclusion criteria have been further elucidated.

Reviewer #2: Introduction

1. The introduction section provides only minimal background information and context. Importantly, there is a lack of awareness or mention of current One Health models in practice, see for example:

1. Rejto N, Ramirez V, Fenelon HT, Xie M, Kuehl K, Terry A, Tin AH, Tabor E, Schneier K, Nee A, Richer A, Rabinowitz P, Meisner J. The One Health Clinic: Care for Young Adults and Companion Animals Experiencing Homelessness. J Prim Care Community Health. 2025 Jan-Dec;16:

2. Aguirre Siliezar K, Patel S, Chande R, Joiner A, Hoover MC, Mathis MW, Hendrickson J, Siliezar J, Jankowski K. Utilizing the Human Animal Bond to Promote Preventive Care Engagement in Underserved Communities: A Descriptive Study of 2 U.S. One Health Clinics. J Prim Care Community Health. 2025 Jan-Dec;16

3. Wagner K, Singletary M, Kohler LN, Ellingson KD, Wagner J, Pogreba-Brown K, Cullen TA. Integrating Human-Animal Care Through a Public Health-Driven One Health Clinic Model in Pima County, Arizona, October 2023-February 2024. Public Health Rep. 2026 Jan-Feb;141(1):31-37.

4. Tin AH, Kuehl K, Terry A, Meisner J, Ramirez V, Tabor E, Rabinowitz P. A One Health Clinic for People Experiencing Homelessness and Their Animals: Treating the Human-Animal Unit. Ann Fam Med. 2022 Sep-Oct;20(5):487.

Thank you for these references, which have been incorporated.

2. In. addition, there appear to be sone random open parenthesis on the last line of the first paragraph We could not locate these

RESEARCH APPROACH

Participant Recruitment

1. I would have liked better details about participant recruitment “#2) Identifying service users who would not have their mental health compromised through participation.” How? My whom? All researchers were veterinarians. We have added a sentence to confirm that the identification was done by the hostel staff, who have extensive experience in working with this population.

2. Snowball sampling through initial research team contacts: Were the Service Users also through previous contacts of research team? Or through the shelter (which wouldn’t be their contacts)? We have clarified this in the manuscript, and refer back to the point above.

3. Tables 1 and 2 could be combined- duplicative

4. Table 3 is too identifiable, sensitive gender information, and the small sample size, even with peudonmyms (is that necessary?) would delete or combine with tables 1 and 2. We thank you for these comments and have given this matter careful consideration. However, we feel that combining the two tables would complicate the data presentation. Furthermore, the use of pseudonyms and number of other people found in each location is, we feel, sufficient to maintain anonymity.

5. Table 1: The term “Veterinary Nurse” is not used in the US. Is that a Veterinary Tech? It would help US readers understand if it was defined, We have added “akin to vet tech” in main manuscript for clarification.

6. Table 2: The abstract seems to focus on dogs, but Table 2 inclusion criteria for service users says “companion animals.” All respondents had dogs. Was that happenstance or did they actually select for dogs. If so, inclusion criteria should be “with dogs.” If not, what if some participants had had cats? Suggest that the authors need to clarify whether other animals were present or did selection criteria exclude them. Thank you. We have clarified that the three participants selected happened to have dogs, whilst in the discussion, noted that although dogs are the most common species found as companion animals to PEH in the UK, other species can also be present.

7. Table 3: The small sample size and selection criteria needs to be considered here- are qualitative data from 1 Trans person and 2 Males based out of one shelter representative of a larger population? It would appear unlikely. We have addressed this in the study limitations.

Ethics and Consent

1. Were any incentives offered to participants for their time and participation in the study? Since dealing with a vulnerable population this should be considered. As stated in the original manuscript and highlighted for clarity -no incentives were offered.

Participant Interviewing

1. Is there bias in response because research RS “provide veterinary support to animal and their humans experiencing homelessness in the UK.” (See “Researcher positionalities”) If the homelessness population is small and many know each other (noted later), will a veterinarian providing care to this community get unbiased responses from service users? RS is a volunteer providing veterinary care in the southwest, the three service users were all in Edinburgh. RS did not volunteer for the duration of this study.

Data Processing and analysis:

1. I am having a hard time with their qualitative methodology due to the small sample size. This reviwer questions whether IPA is an appropriate analytic method given the sample size, and what they are trying to force into that box. They write:

“Interpretative Phenomenological Analysis (IPA) was selected for data analysis as best

suited for exploring peoples’ lived experiences of a specific phenomenon [10]. IPA’s

double hermeneutic approach, in which participants provide their own interpretation of

their experience which the researcher in turn interprets, is considered appropriate for

‘topics which are complex, ambiguous and emotionally laden’ [11]. The number of

participants in this study (n=13) exceeded that typically used for an IPA project (usually

<10). Small sample sizes are desirable to enable the detailed idiographic analysis of

lived experience using IPA. However larger sample sizes (as here) avoid the risk of loss

of detail when interviews are tightly focused around a central research question or

when individuals share certain important characteristics, in this case the common

experience of homelessness [10].”

• There are only 3 PEH interviews, 2 Males and 1 Tran person, all with dogs, coming from one shelter. Again, this seems unlikely to be full representative of the greater population in the 6 UK locations identified (page 4)

This is acknowledged in the study limitations: The number of service users was small, however this is a benefit for IPA based studies, with Smith el al (2022) recommending n=3-10. Participants are selected to be representative of a perspective, rather than a population. This study was a multi-perspective study, comparing and contrasting the IPA of service providers and service users, in order to gain a broad and holistic understanding of the healthcare needs and challenges of the specific population studied here.

• There are 13 respondents, but only 3 PEH respondents. The authors state that their respondent size is a positive to “avoid the risk of loss of detail” but they are lumping the 3 PEH into the group of healthcare providers, and also healthcare providers with housing services and animal welfare. This could potentially dilute the findings for any particular subgroup.

See above -the two groups are not lumped together, they are treated as two linked groups, linked through the phenomenon of homelessness. Multi-perspective studies are a recognised use of IPA, in order to gain “ a multi-faceted account of the phenomenon” (Smith et al 2022).

• I think this is an informative study, but I don’t know that IPA makes sense.

We appreciate the question but disagree as IPA has been proven an excellent choice of methodology for providing a qualitative study of complex emotional subjects within a small sample cohort. •

Results

Research Question 1:

Healthcare Needs

1. There is reference to Spay/Neuter challenges, but this is not referenced again in the text. Suggest either expanding or deleting. This has been deleted.

2. Return to later Sally (GP)’s quote….are there community vets who say the same thing? I mean, it’s the case in housed families too. I’m not sure what this refers to?

3. Is the ransom piece necessary? Will a joint clinic influence this? Why is this under “Animal healthcare”?. This statement has been deleted.

4. Page 13, section iii : “According to service providers, every PEH they encountered also had substance abuse concerns related to previous trauma.” To state that every had this concern appears to be an exaggeration, and appears pejorative. This was the statement of every single service provider and so not pejorative with respect to being an accurate reflection of their interview stat

Attachment Submitted filename: BECKYR~1.DOC

# Peer review: Decision Letter 1

**DOI:** 10.1371/journal.pone.0354517.r003

**Authors:** Ulrike Gertrud Munderloh

14 May 2026

Dear Dr. Cousquer,

Thank you for submitting your manuscript to PLOS ONE. After careful consideration, we feel that it has merit but does not fully meet PLOS ONE’s publication criteria as it currently stands. Therefore, we invite you to submit a revised version of the manuscript that addresses the points raised during the review process.

Please provide interpretations as requested by reviewer 3.

Please submit your revised manuscript by Jun 28 2026 11:59PM. If you will need more time than this to complete your revisions, please reply to this message or contact the journal office at plosone@plos.org. When you're ready to submit your revision, log on to https://www.editorialmanager.com/pone/ and select the 'Submissions Needing Revision' folder to locate your manuscript file.

- A letter that responds to each point raised by the academic editor and reviewer(s). You should upload this letter as a separate file labeled 'Response to Reviewers'.
- A marked-up copy of your manuscript that highlights changes made to the original version. You should upload this as a separate file labeled 'Revised Manuscript with Track Changes'.
- An unmarked version of your revised paper without tracked changes. You should upload this as a separate file labeled 'Manuscript'.

If you would like to make changes to your financial disclosure, please include your updated statement in your cover letter. Guidelines for resubmitting your figure files are available below the reviewer comments at the end of this letter.

If applicable, we recommend that you deposit your laboratory protocols in protocols.io to enhance the reproducibility of your results. Protocols.io assigns your protocol its own identifier (DOI) so that it can be cited independently in the future. For instructions see: https://journals.plos.org/plosone/s/submission-guidelines#loc-laboratory-protocols. Additionally, PLOS ONE offers an option for publishing peer-reviewed Lab Protocol articles, which describe protocols hosted on protocols.io. Read more information on sharing protocols at https://plos.org/protocols?utm_medium=editorial-email&utm_source=authorletters&utm_campaign=protocols.

As the corresponding author, your ORCID iD is verified in the submission system and will appear in the published article. PLOS supports the use of ORCID, and we encourage all coauthors to register for an ORCID iD and use it as well. Please encourage your coauthors to verify their ORCID iD within the submission system before final acceptance, as unverified ORCID iDs will not appear in the published article. *Only*  the individual author can complete the verification step; PLOS staff *cannot*  verify ORCID iDs on behalf of authors.

We look forward to receiving your revised manuscript.

Kind regards,

Ulrike Gertrud Munderloh, Ph.D.

Academic Editor

PLOS One

Journal Requirements:

If the reviewer comments include a recommendation to cite specific previously published works, please review and evaluate these publications to determine whether they are relevant and should be cited. There is no requirement to cite these works unless the editor has indicated otherwise.

Please review your reference list to ensure that it is complete and correct. If you have cited papers that have been retracted, please include the rationale for doing so in the manuscript text, or remove these references and replace them with relevant current references. Any changes to the reference list should be mentioned in the rebuttal letter that accompanies your revised manuscript. If you need to cite a retracted article, indicate the article’s retracted status in the References list and also include a citation and full reference for the retraction notice.

[Note: HTML markup is below. Please do not edit.]

Reviewers' comments:

Reviewer's Responses to Questions

**Comments to the Author**

Reviewer #1: All comments have been addressed

Reviewer #3: (No Response)

**********

2. Is the manuscript technically sound, and do the data support the conclusions?

Reviewer #1: Yes

Reviewer #3: Yes

**********

3. Has the statistical analysis been performed appropriately and rigorously?

Reviewer #1: N/A

Reviewer #3: N/A

**********

4. Have the authors made all data underlying the findings in their manuscript fully available?

The PLOS Data policy

Reviewer #1: No

Reviewer #3: Yes

**********

5. Is the manuscript presented in an intelligible fashion and written in standard English?

Reviewer #1: Yes

Reviewer #3: Yes

**********

Reviewer #1: Thank you for your thoughtful revisions and your careful attention to the initial reviewer comments. The expanded background and discussion sections now provide important context for situating the current study within the broader body of research, as well informing directions for future work in this area. In addition, the added clarity in the exclusion criteria and limitations enhances the transparency, reproducibility, and interpretation of your findings. The rational and process described for data availability seems appropriate for the sensitive nature and type of data collected for this study.

I find that all of my prior concerns have been adequately addressed, and I have no further recommendations at this time.

Reviewer #3: 1) The abstract seems very long and like a thesis abstract. It should be condensed

2) I personally would not use dot point in the method

3) The results are very thin. While the themes seem reasonable, the data presented is accompanied by little interpretation. Themes should never end on a quote that is not interpreted

4) I also wouldn't use dot points to summarise subthemes

**********

what does this mean?). If published, this will include your full peer review and any attached files.

If you choose “no”, your identity will remain anonymous but your review may still be made public.

**Do you want your identity to be public for this peer review?**  For information about this choice, including consent withdrawal, please see our Privacy Policy

Reviewer #1: No

Reviewer #3: **Yes:**  Damien W Riggs

**********

[NOTE: If reviewer comments were submitted as an attachment file, they will be attached to this email and accessible via the submission site. Please log into your account, locate the manuscript record, and check for the action link "View Attachments". If this link does not appear, there are no attachment files.]

To ensure your figures meet our technical requirements, please review our figure guidelines: https://journals.plos.org/plosone/s/figures

You may also use PLOS’s free figure tool, NAAS, to help you prepare publication quality figures: https://journals.plos.org/plosone/s/figures#loc-tools-for-figure-preparation.

NAAS will assess whether your figures meet our technical requirements by comparing each figure against our figure specifications.

# Author comment: Author response to Decision Letter 2

**DOI:** 10.1371/journal.pone.0354517.r004

1 Jul 2026

Good afternoon,

Thank you kindly for these further comments and for the considerable attention to detail. We are delighted that the vast majority of queries raised have been addressed to the satisfaction of the reviewers.

We have now reviewed the clean copy of the manuscript that was submitted for re-review in mid-February and cross checked it against the comments from the reviewers. There are 4 outstanding points that we can see, which we have copied and responded to here. This has allowed us to produce the minor revision with edits marked in yellow on the attached file.

The following is a summary of our response to Reviewer #3:

(1) The abstract seems very long and like a thesis abstract. It should be condensed.

Reply: The abstract is 265 words, which seems well within the standard 150-300 word limits for scientific journals. There are no specific Plos One abstract length guidelines and we, the authors, feel that this abstract cannot be further shortened, without losing key information and the other reviewer has not found this to be an issue.

(2) I personally would not use dot point in the method.

Reply: We assume by ‘dot point’, you are referring to our use of bullet points. These bullet points are used to identify key sentences that stand out from the main body of text. As this comment is a "personal preference" of the reviewer and not consistent across reviewers, we ask that this suggestion be waived.

3) The results are very thin. While the themes seem reasonable, the data presented is accompanied by little interpretation. Themes should never end on a quote that is not interpreted.

Reply: We thank the reviewer for this observation and acknowledge that, to avoid an overly long manuscript, we have had to reduce an initial qualitative thesis of over 15,000 word down to the most salient quotes, that in most cases speak for themselves, with key surrounding sentences to provide interpretative context. Where a theme ended on a quote, a paragraph of interpretation has now been added (highlighted in yellow text on attached manuscript). Thank you for highlighting that this would be helpful to the reader. It is perhaps worth noting that the original work is full of qualitatively rich material and the resulting insights are, we believe, rich rather than thin. The challenge of writing about the themes and subthemes is significant and we are aware that some readers may want material interpreted whereas others will want to be allowed more freedom to consider the proposed interpretations.

(4) I also wouldn't use dot points to summarise subthemes.

Reply: Please see response to 2) above.

Attachment Submitted filename: BECKYR~1_auresp_2.DOC

# Peer review: Decision Letter 2

**DOI:** 10.1371/journal.pone.0354517.r005

**Authors:** Ulrike Gertrud Munderloh

10 Jul 2026

‘My dog is my life’:

One Health approaches to healthcare for people and their companion animals experiencing homelessness in the United Kingdom

PONE-D-25-51060R2

Dear Dr. Cousquer,

We’re pleased to inform you that your manuscript has been judged scientifically suitable for publication and will be formally accepted for publication once it meets all outstanding technical requirements.

Within one week, you’ll receive an e-mail detailing the required amendments. When these have been addressed, you’ll receive a formal acceptance letter and your manuscript will be scheduled for publication.

An invoice will be generated when your article is formally accepted. Please note, if your institution has a publishing partnership with PLOS and your article meets the relevant criteria, all or part of your publication costs will be covered. Please make sure your user information is up-to-date by logging into Editorial Manager at Editorial Manager® and clicking the ‘Update My Information' link at the top of the page. For questions related to billing, please contact billing support.

If your institution or institutions have a press office, please notify them about your upcoming paper to help maximize its impact. If they’ll be preparing press materials, please inform our press team as soon as possible -- no later than 48 hours after receiving the formal acceptance. Your manuscript will remain under strict press embargo until 2 pm Eastern Time on the date of publication. For more information, please contact onepress@plos.org.

Kind regards,

Ulrike Gertrud Munderloh, Ph.D.

Academic Editor

PLOS One

Additional Editor Comments (optional):

Reviewers' comments:

Reviewer's Responses to Questions

**Comments to the Author**

Reviewer #3: All comments have been addressed

**********

2. Is the manuscript technically sound, and do the data support the conclusions?

Reviewer #3: Yes

**********

3. Has the statistical analysis been performed appropriately and rigorously?

Reviewer #3: N/A

**********

4. Have the authors made all data underlying the findings in their manuscript fully available?

The PLOS Data policy

Reviewer #3: No

**********

5. Is the manuscript presented in an intelligible fashion and written in standard English?

Reviewer #3: Yes

**********

Reviewer #3: Given the authors acknowledge this is student work, I would have liked to see a less defensive and more engaged response.

**********

what does this mean?). If published, this will include your full peer review and any attached files.

If you choose “no”, your identity will remain anonymous but your review may still be made public.

**Do you want your identity to be public for this peer review?**  For information about this choice, including consent withdrawal, please see our Privacy Policy

Reviewer #3: No

**********

# Editor report: Acceptance letter

**DOI:** 10.1371/journal.pone.0354517.r006

**Authors:** Ulrike Gertrud Munderloh

PONE-D-25-51060R2

PLOS One

Dear Dr. Cousquer,

I'm pleased to inform you that your manuscript has been deemed suitable for publication in PLOS One. Congratulations! Your manuscript is now being handed over to our production team.

At this stage, our production department will prepare your paper for publication. This includes ensuring the following:

* All references, tables, and figures are properly cited

* All relevant supporting information is included in the manuscript submission,

* There are no issues that prevent the paper from being properly typeset

You will receive further instructions from the production team, including instructions on how to review your proof when it is ready. Please keep in mind that we are working through a large volume of accepted articles, so please give us a few days to review your paper and let you know the next and final steps.

Lastly, if your institution or institutions have a press office, please let them know about your upcoming paper now to help maximize its impact. If they'll be preparing press materials, please inform our press team within the next 48 hours. Your manuscript will remain under strict press embargo until 2 pm Eastern Time on the date of publication. For more information, please contact onepress@plos.org.

You will receive an invoice from PLOS for your publication fee after your manuscript has reached the completed accept phase. If you receive an email requesting payment before acceptance or for any other service, this may be a phishing scheme. Learn how to identify phishing emails and protect your accounts at https://explore.plos.org/phishing.

If we can help with anything else, please email us at customercare@plos.org.

Thank you for submitting your work to PLOS One and supporting open access.

Kind regards,

PLOS One Editorial Office Staff

on behalf of

Dr. Ulrike Gertrud Munderloh

Academic Editor

PLOS One
